# Activation of the c-Jun NH_2_-terminal kinase pathway by coronavirus infectious bronchitis virus promotes apoptosis independently of c-Jun

**DOI:** 10.1038/s41419-017-0053-0

**Published:** 2017-12-13

**Authors:** To Sing Fung, Ding Xiang Liu

**Affiliations:** 1 South China Agricultural University, Guangdong Province Key Laboratory Microbial Signals & Disease Co, and Integrative Microbiology Research Centre, Guangzhou, 510642 Guangdong, People’s Republic of China; 2 School of Biological Sciences, Nanyang Technological University, 60 Nanyang Drive, Singapore, 63755 Singapore

## Abstract

Mitogen-activated protein kinases (MAPKs) are conserved protein kinases that regulate a variety of important cellular signaling pathways. Among them, c-Jun N-terminal kinases (JNK) are known to be activated by various environmental stresses including virus infections. Previously, activation of the JNK pathway has been detected in cells infected with several coronaviruses. However, detailed characterization of the pathway as well as its implication in host–virus interactions has not been fully investigated. Here we report that the JNK pathway was activated in cells infected with the avian coronavirus infectious bronchitis virus (IBV). Of the two known upstream MAPK kinases (MKK), MKK7, but not MKK4, was shown to be responsible for IBV-induced JNK activation. Moreover, knockdown and overexpression experiments demonstrated that JNK served as a pro-apoptotic protein during IBV infection. Interestingly, pro-apoptotic activity of JNK was not mediated via c-Jun, but involved modulation of the anti-apoptotic protein B-cell lymphoma 2 (Bcl2). Taken together, JNK constitutes an important aspect of coronavirus–host interaction, along with other MAPKs.

## Introduction

Mitogen-activated protein kinases (MAPKs) are conserved kinases regulating critical signaling pathways, such as apoptosis, differentiation, and immune response^1^. So far, four subgroups of MAPKs are identified, namely extracellular regulated kinase 1/2 (ERK1/2), ERK5, p38, and c-Jun N-terminal kinases (JNK)^2, 3^. Among them, ERK1/2 is activated by growth factors and mitogens, whereas p38 and JNK respond to cellular stresses and/or environmental stimuli^2^. MAPKs are activated by kinase cascades. In particular, JNK is activated by MAPK kinases 4(MKK4) or MKK7, which are phosphorylated by upstream MAPK kinase kinases^4^. JNK activation requires dual phosphorylation of Thr and Tyr within a conserved Thr-Pro-Tyr motif^4^. Active JNK phosphorylates c-Jun and other substrates to modulate their activities^5^. For example, phosphorylated c-Jun dimerizes with other transcription factors to form activator protein-1(AP-1) complex, thereby activating transcription of target genes^6^.

JNK pathway modulates apoptosis by two mechanisms: transactivation of pro-apoptotic genes and interactions with B-cell lymphoma 2 (Bcl2) family proteins^7^. JNK-dependent activation of AP-1 upregulates expression of pro-apoptotic genes such as Bcl2 homologous antagonist killer, Fas ligand, and tumor necrosis factor-alpha^8^. Some transcription factors, such as p53 and p73, are also activated by JNK and promote cell death^9, 10^. Also, JNK can translocate into mitochondria and modulate the function of Bcl2 family proteins, such as BH3-interacting domain death agonist^11^, Bcl2-interacting mediator of cell death^12^, and Bcl2-associated death promoter^13, 14^. JNK can also directly phosphorylate Bcl2, inhibiting its anti-apoptotic activity^15^.

MAPK activation is observed during coronavirus infection, modulating various aspects of virus–host interaction^16^. For example, p38 phosphorylation during IBV infection upregulates the expression of pro-inflammatory cytokines interleukin 6 (IL-6) and IL-8^17^, while ERK1/2 is activated and plays a pro-survival role in ER stress-induced apoptosis during IBV infection^18^. JNK phosphorylation was detected in cells infected with MHV or SARS-CoV^19, 20^, and in cells overexpressing the N, 3a, 3b, or 7a protein of SARS-CoV^21–24^. JNK and Akt are required for establishing persistent SARS-CoV infection^25^. In cells overexpressing the SARS-CoV spike protein, JNK phosphorylation is mediated by protein kinase C epsilon^26^, and the expression of IL-8 depends on the activity of AP-1^27^. However, detailed mechanisms of JNK activation during coronavirus infection and its involvement in coronavirus-induced apoptosis are largely unknown.

Previously, we showed that ER stress sensor inositol-requiring enzyme 1 (IRE1) protects cells from IBV-induced apoptosis partly by modulating JNK phosphorylation^28^. Here we determined upstream MKKs of IBV-induced JNK activation and characterized its involvement in regulating IBV-induced apoptosis. We found that IBV infection activated the MKK7/JNK/c-Jun pathway in two mammalian cells (H1299 and Huh-7). IBV-induced JNK activation was mediated by MKK7, and required both its ATP binding and phosphorylation sites. We also showed that JNK-promoted apoptosis during IBV infection, and this activity was not mediated via c-Jun, but involved modulation of Bcl2. Taken together, our data demonstrate an important pro-apoptotic function of JNK during coronavirus infection.

## Results

### IBV infection activates the MKK7-JNK-Jun pathway

**Fig. 1:**
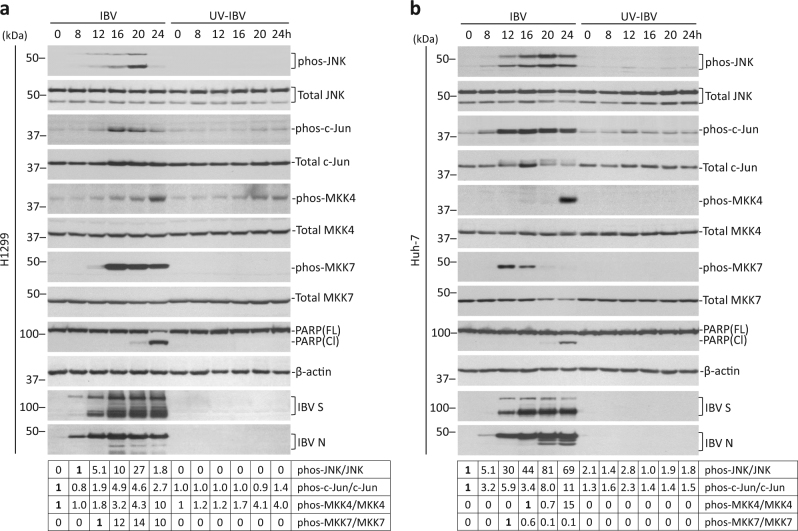
IBV infection activated the JNK signaling pathway **a** Activation of JNK pathway during IBV infection in H1299 cells. H1299 cells were infected with IBV at MOI~2 or were mock infected. Protein lysates were harvested at the indicated time points and were subjected to Western blot analysis using the indicated antibodies. Beta-actin was included as loading control. Sizes of protein ladders in kDa were indicated on the left. Degree of protein phosphorylation was calculated as the band intensity of phosphorylated protein divided by the band intensity of the total protein, respectively. The experiment was repeated three times with similar results, and the result of one representative experiment is shown. **b** Activation of JNK pathway during IBV infection in Huh-7 cells. Infection, Western blot analysis and quantification were performed as in **a**. The experiment was repeated three times with similar results, and the result of one representative experiment is shown.

Activation of JNK pathway was determined in H1299 cells. Total JNK remained unchanged in both IBV-infected cells and UV-IBV control (Fig. 1a). Phosphorylated JNK (phos-JNK) first appeared at 12 hpi, peaked at 20 hpi, and rapidly disappeared to background level at 24 hpi in IBV-infected cells. No phos-JNK was observed in UV-IBV control. A low level of phosphorylated c-Jun (phos-c-Jun) was detected in UV-IBV control and in early IBV-infected samples, possibly due to basal activation or nonspecific detection. A sudden increase of phos-c-Jun was observed at 16 hpi, which slowly subsided later. Total c-Jun was slightly higher at 16–24 hpi compared to early infected samples or UV-IBV control, indicating that phosphorylation may stabilize c-Jun. Total MKK4 and MKK7 remained unchanged in both IBV-infected and UV-IBV control. A basal level of phosphorylated MKK4 (phos-MKK4) was detected at 0 hpi, which gradually increased and peaked at 24 hpi. Phos-MKK4 also stably accumulated in UV-IBV control, but remained lower than infected samples of the same time point. No detectable phos-MKK7 was observed in UV-IBV control, but a drastic increase of phos-MKK7 was detected at 16 hpi, which slowly decreased, but remained high even at 24 hpi.

In Huh-7 cells, IBV infection induced similar JNK phosphorylation, though phos-JNK signal was stronger and remained high even at 24 hpi (Fig. 1b). Significant c-Jun phosphorylation was also detected in IBV-infected Huh-7 cells, but phosphorylation seemed to destabilized c-Jun, as total c-Jun reduced at 20–24 hpi. Minimum phos-MKK4 was observed, except in 24 h-infected sample. Phos-MKK7 appeared earlier at 12 hpi, but diminished faster than in H1299 cells. Phos-MKK7 could be hardly detected at 20–24 hpi, possibly also due to reduction of total MKK7. Taken together, JNK pathway was activated during IBV infection and JNK was more likely phosphorylated by MKK7 instead of MKK4. Notably, phos-JNK preceded PARP cleavage in IBV-infected cells, suggesting its potential involvement in regulating apoptosis (Fig. 1a, b).

### Overexpression of MKK7 promotes JNK phosphorylation during IBV infection

**Fig. 2:**
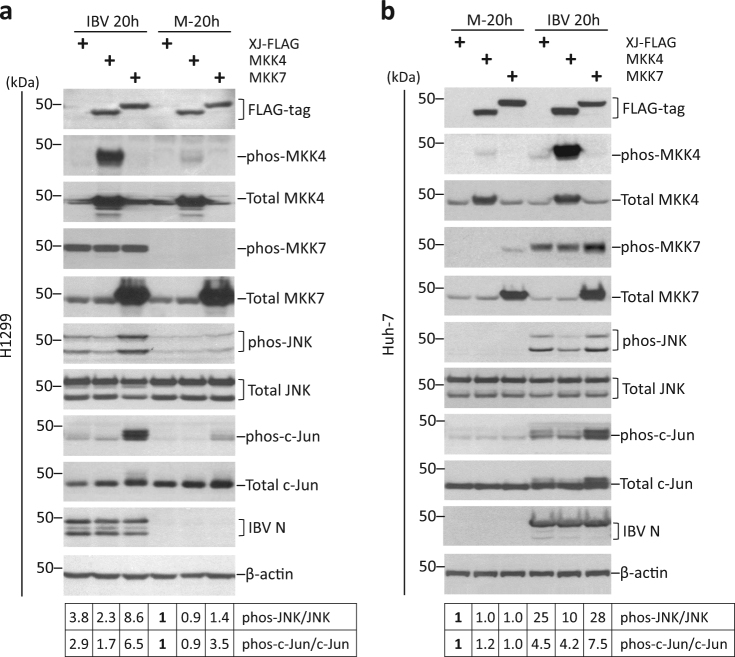
Overexpression of MKK7 promotes IBV-induced JNK phosphorylation **a** Overexpressing MKK7 promotes JNK/c-Jun phosphorylation in H1299 cells. H1299 cells were transfected with pXJ40FLAG, pXJ40FLAG-MKK4, or pXJ40FLAG-MKK7 before being infected with IBV or being mock infected for 20 h. Protein lysates were harvested at the indicated time points and were subjected to Western blot analysis using the indicated antibodies. Beta-actin was included as loading control. Sizes of protein ladders in kDa were indicated on the left. Degree of protein phosphorylation was calculated as in Fig. 1a. The experiment was repeated three times with similar results, and the result of one representative experiment is shown. **b** MKK7 promotes the activation of JNK pathway in Huh-7 cells. Transfection and infection of Huh-7 cells, Western blot, and quantification were performed as in **a**. The experiment was repeated three times with similar results, and the result of one representative experiment is shown.

To determine which MKK activates JNK, H1299 cells were transfected with MKK4 or MKK7 plasmids before infected with IBV. FLAG-tag MKK4 and MKK7 were determined by Western blot (Fig. 2a). As expected, phos-MKK4 was significantly higher in infected cells transfected with FLAG-MKK4, compared with vector-transfected control (XJ-FLAG). Surprisingly, phos-MKK7 was detected at similar level in all infected cells, although a much higher level of total MKK7 was observed in FLAG-MKK7-transfected cells. It was possible that phos-MKK7 antibody only recognized endogenous, but not ectopically expressed phos-MKK7. Surprisingly, MKK4 overexpression slightly reduced IBV-induced phosphorylation of JNK and c-Jun. In contrast, both phos-JNK and phos-c-Jun were detected at much higher levels in FLAG-MKK7-transfected IBV-infected cells, compared with vector control. Phos-JNK and phos-c-Jun in mock-infected cells were also slightly higher in FLAG-MKK7-transfected cells. Transfection of MKK4 or MKK7 did not affect IBV replication, as similar IBV N protein was detected compared with vector control.

Compared to vector control, a slightly higher phos-MKK7 was detected in FLAG-MKK7-transfected Huh-7 cells (Fig. 2b). Similarly, although ectopically expressed FLAG-MKK4 was highly phosphorylated, it actually reduced IBV-induced JNK phosphorylation compared with vector control. Although phos-JNK was only marginally increased in FLAG-MKK7-tranfected IBV-infected cells, phos-c-Jun level was significantly higher than vector control. Thus, overexpressed FLAG-MKK7 was functionally active and promoted phosphorylation of JNK and c-Jun induced by IBV infection.

### Overexpression of MKK7 promotes IBV-induced apoptosis

**Fig. 3:**
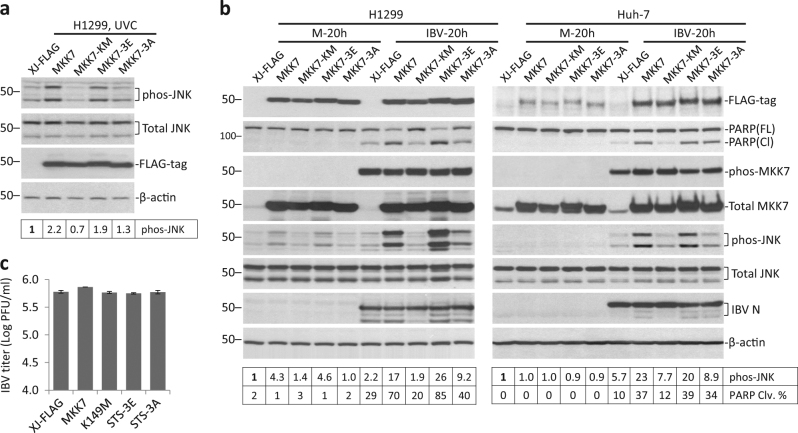
Overexpression of MKK7 or its phosphomimetic promotes IBV-induced apoptosis **a** H1299 cells were transfected with pXJ40-FLAG, pXJ40FLAG-MKK7, pXJ40FLAG-MKK7-KM, pXJ40FLAG-MKK7-3E, or pXJ40FLAG-MKK7-3A. After 24 h, the cells were irradiated with UVC (10 mJ) and incubated for another 1 h. Protein lysates were harvested and were subjected to Western blot analysis using the indicated antibodies. Beta-actin was included as loading control. Sizes of protein ladders in kDa were indicated on the left. Degree of JNK phosphorylation was calculated as in Fig. 1a. The experiment was repeated three times with similar results, and the result of one representative experiment is shown. **b** H1299 or Huh-7 cells were transfected as in **a** before being infected with IBV at MOI~2 or being mock infected for 20 h. The cells were harvested and were subjected to Western blot analysis using the indicated antibodies. Beta-actin was included as loading control. Degree of JNK phosphorylation was calculated as in Fig. 1a. Percentage of PARP cleavage [PARP Clv. (%)] was calculated as the intensity of cleaved PARP [PARP(Cl)] divided by the total intensities of full length PARP [PARP(FL)] and PARP(Cl).The experiment was repeated three times with similar results, and the result of one representative experiment is shown. **c** The culture supernatants of IBV-infected Huh-7 cells in **b** were subjected to plaque assay analysis. Virus titers were expressed as the logarithm of plaque-forming units (PFU) per ml of supernatants.

As JNK activation is associated with IBV-induced apoptosis, we then tested the effect of MKK7 overexpression on apoptosis. Also, to determine the functional domain required, several MKK7 mutants were generated. ATP-binding site (K149) was mutated in the KM mutant. Residues phosphorylated by MKKKs (S271, T275, and S277) were mutated to glutamates in the 3E mutant, which served as a “phosphomimetic” of active MKK7. In the 3A mutant, the same three residues were mutated to alanines, rendering it resistant to phosphorylation by upstream kinases. Transfected H1299 cells were irradiated with UVC, a condition known to activate JNK pathway. UVC-induced moderate JNK phosphorylation in vector control (Fig. 3a). Wild-type MKK7-enhanced UVC-induced JNK phosphorylation by ~2.2-fold. Phos-JNK in MKK7-KM-transfected cells was slightly lower than vector control, so it might serve as a dominant negative mutant that inhibited activation of endogenous MKK7. Unexpectedly, although previously treated as constitutively active, the 3E mutant promoted UVC-induced JNK activation only similarly as wild-type MKK7. Also, unlike KM mutant, MKK7-3A-transfected cells had similar phos-JNK level as vector control, indicating that MKK7-3A was not dominant negative.

Similarly transfected H1299 cells were infected with IBV. Expression of wildtype and mutant MKK7 were determined by Western blot (Fig. 3b). Phos-MKK7 was detected at similar level in all IBV-infected cells, but not in mock-infected cells. Because phosphorylation sites were removed in the MKK7-3A mutant, phos-MKK7 detected in MKK7-3A-transfected cells should only represent endogenous phos-MKK7. Thus, it was concluded that phos-MKK7 antibody indeed only detected endogenous phos-MKK7, but not ectopically expressed protein. Notably, transfection of wild-type MKK7 or 3E significantly increased phos-JNK level in IBV-infected cells, as compared with vector control. Transfection of the KM mutant slightly reduced JNK phosphorylation. In MKK7-3A-transfected IBV-infected cells, phos-JNK level was higher than vector control, but lower than in cells transfected with wild-type MKK7 or 3E. PARP cleavage percentages correlated well with JNK phosphorylation: moderate PARP cleavage was detected in vector control, which was slightly reduced in KM, slightly increased in 3A, and considerably increased in cells transfected with wild-type MKK7 or 3E.

In Huh-7 cells, IBV-induced phos-MKK7 was slightly higher in cells transfected with wild-type MKK7, KM, or 3A. Nonetheless, phos-JNK showed a similar pattern as in H1299 cells: only wild-type MKK7 and 3E, but not KM or 3A, significantly enhanced IBV-induced JNK phosphorylation compared with vector control (Fig. 3b). PARP cleavage pattern also correlated with JNK activation, same as in H1299 cells. As IBV N was detected at slightly different levels in the transfected cells, plaque assay was performed to titrate IBV in the supernatants of infected cells. As shown in Fig. 3c, similar IBV titers were detected in all transfected samples, suggesting that transfection of MKK7 or mutants did not affect IBV replication in Huh-7 cells. Therefore, both ATP binding and phosphorylation sites were required for MKK7 to activate JNK during IBV infection. Moreover, MKK7-dependent JNK phosphorylation promoted IBV-induced apoptosis.

### Overexpression of constitutively active JNK promotes IBV-induced apoptosis

**Fig. 4:**
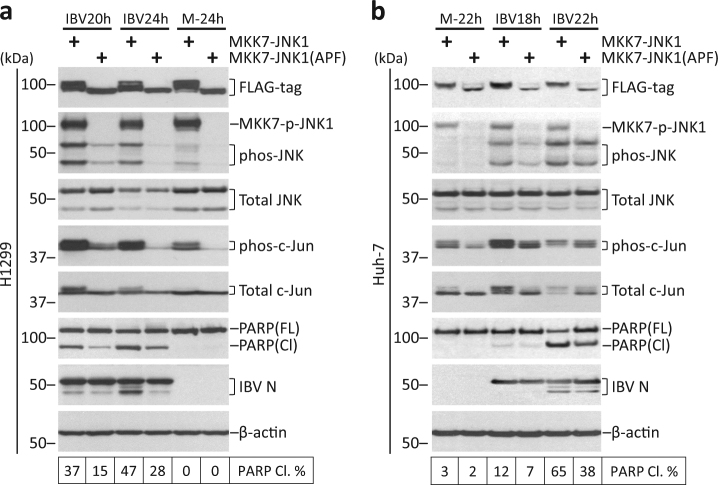
Overexpression of constitutively active JNK promotes IBV-induced apoptosis **a** H1299 cells in duplicate were transfected with pcDNA-MKK7-JNK1 or pcDNA-MKK7-JNK1(APF), before being infected with IBV at MOI~2 or being mock infected. One set of cells were harvested for protein at the indicated time points and were subjected to Western blot analysis using the indicated antibodies. Beta-tubulin was included as loading control. Sizes of protein ladders in kDa were indicated on the left. Degree of JNK phosphorylation and the percentage of PARP cleavage was determined as in Fig. 3b. The experiment was repeated three times with similar results, and the result of one representative experiment is shown. **b** Huh-7 cells were transfected and infected similarly as in **a.** Western blot analysis and data quantification were performed as in **a**. The experiment was repeated three times with similar results, and the result of one representative experiment is shown.

Fusion of JNK to MKK7 renders the protein constitutively active^29^. As a negative control, conserved Thr-Pro-Tyr motif in JNK was modified to Ala-Pro-Phe (APF), rendering it unable to be phosphorylated. H1299 cells were transfected with pcDNA-FLAG-MKK7-JNK1 or pcDNA-FLAG-MKK7-JNK1(APF), before infected with IBV or mock infected. Expression of MKK7-JNK1 was slightly higher than APF, possibly due to differences in protein stability (Fig. 4a). JNK1 within the fusion protein was efficiently phosphorylated in MKK7-JNK1-transfected cells, but not in the APF mutant, as determined by blotting with phos-JNK antibody. Notably, endogenous phos-JNK was promoted in MKK7-JNK1-transfeced cells compared to APF control, while endogenous total JNK was not affected. Compared to APF control, transfection of MKK7-JNK1 also increased phos-c-Jun in both mock-infected and IBV-infected cells, indicating that MKK7- JNK1 could indeed activate the downstream pathway. PARP cleavage was found significantly higher in cells transfected with MKK7-JNK1, as compared with APF control. Expression of constitutively active JNK did not affect IBV replication, as IBV N level was similar to the APF control.

Transfection of MKK7-JNK1 had a similar effect on the phosphorylation of endogenous JNK and c-Jun in Huh-7 cells (Fig. 4b). Also, a significantly higher percentage of PARP cleavage was detected at 24 hpi in Huh-7 cells transfected with MKK7-JNK1 compared with APF control. Taken together, overexpression of constitutively active JNK also promoted IBV-induced apoptosis.

### SP600125 suppresses JNK phosphorylation and apoptosis during IBV infection

**Fig. 5:**
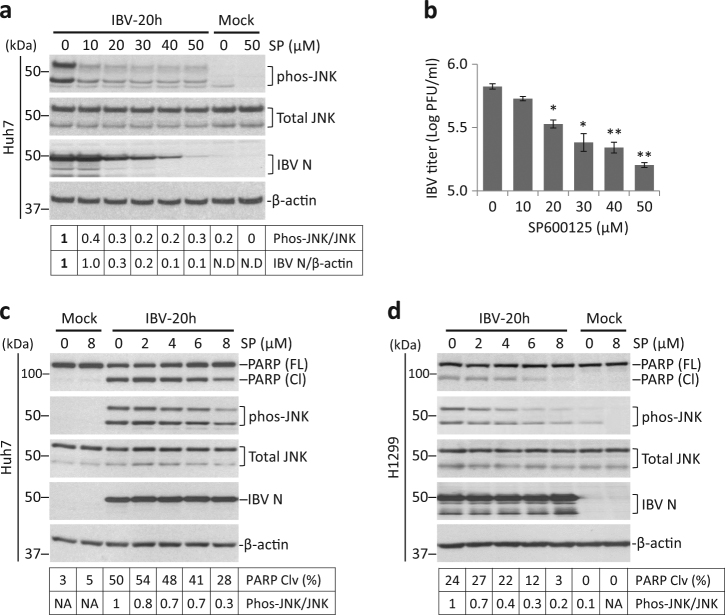
SP600125 suppresses IBV-induced JNK phosphorylation and apoptosis **a** Huh-7 cells were infected with IBV or were mock infected. After 4 h, the cells were treated with SP600125 (SP) at the indicated concentrations or same volume of DMSO for 20 h. Cell lysates were subjected to Western blot analysis using the indicated antibodies. JNK phosphorylation was determined as in Fig. 1a. **b** The culture supernatants in **a** were subjected to plaque assay analysis. Virus titers were expressed as the logarithm of plaque-forming units (PFU) per ml of supernatants. Using Student’s *t* test (two-tailed distribution and unequal variance) to analyze the data, asterisks indicated significant difference compared with DMSO control(*, *p* < 0.05, **, *p* < 0.01). **c** Huh-7 cells were infected and treated with SP600125 at lower concentrations as in **a.** Western blot analysis and data quantification were performed as in **a.** The experiment was repeated three times with similar results, and the result of one representative experiment is shown. **d** H1299 cells were infected and treated with SP600125 as in **c**. Western blot analysis and data quantification were performed as in **a**. The experiment was repeated three times with similar results, and the result of one representative experiment is shown.

SP600125 is a widely used JNK inhibitor^30^. SP600125 treatment was first attempted in Huh-7 cells, which had a more intense IBV-induced JNK phosphorylation. Huh-7 cells were infected with IBV for 4 h before treated with increasing doses of SP600125 for 20 h. IBV infection induced JNK phosphorylation in the solvent control (0 µM). Treatment of 10 μM SP600125 already reduced phos-JNK by ~60%, but higher concentration did not further reduce phos-JNK level (Fig. 5a). This low level of phos-JNK might result from other SP600125-resistant mechanisms. SP600125 treated at 10 μM did not significantly affect IBV N level compared with solvent control, but at higher concentration, it dosage-dependently reduced IBV N protein. Consistently, treatment of SP600125 at 20 μM or above significantly reduced IBV titers in Huh-7 cells (Fig. 5b). Thus, SP600125 inhibited IBV replication at concentration higher than 10 μM.

A lower concentration of SP600125 was used in subsequent experiments. Huh-7 and H1299 cells were infected and treated with 0–8 μM SP600125. Protein levels of total JNK and IBV N was not significantly affected by SP600125 (Fig. 5c, d). Notably, phos-JNK and PARP cleavage both reduced dosage-dependently with increasing concentration of SP600125 in both Huh-7 and H1299 cells. In summary, when treated at <8 µM, JNK inhibition by SP600125 suppressed IBV-induced apoptosis without significantly affecting viral replication.

### Knockdown of JNK, but not c-Jun, attenuates IBV-induced apoptosis

**Fig. 6:**
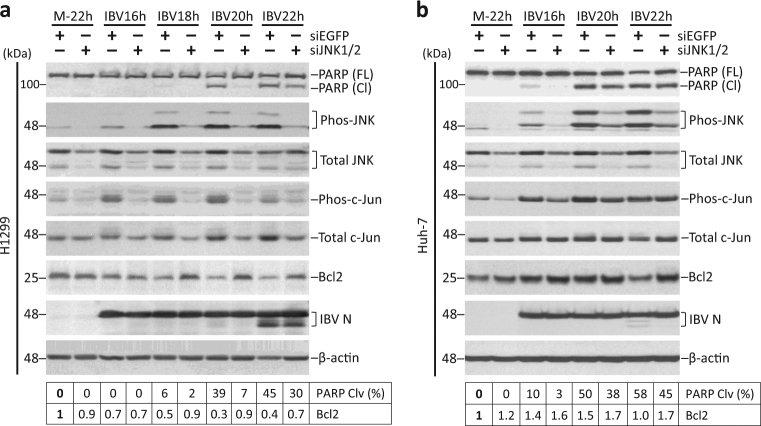
Knockdown of JNK attenuates IBV-induced apoptosis **a** H1299 cells were transfected with siEGFP or siJNK before being infected with IBV at MOI~2 or being mock infected. Cells were harvested at indicated time points and were subjected to Western blot analysis using the indicated antibodies. Beta-actin was included as loading control. Percentage of PARP cleavage was determined as in Fig. 3b. Relative amount of Bcl2 was determined as the band intensity normalized to β-actin, with the mock-infected sample of siEGFP-transfected cells set as one. The experiment was repeated three times with similar results, and the result of one representative experiment is shown. **b** Huh-7 cells were transfected and infected as in **a**. The cells were harvested and were subjected for Western blot analysis as in **a.** Percentage of PARP cleavage and the relative abundance of Bcl2 was determined as in **a.** The experiment was repeated three times with similar results, and the result of one representative experiment is shown.

RNAi approach was adopted to confirm result from the inhibitor experiment. H1299 cells transfected with siEGFP or siJNK were infected with IBV or mock infected. Both phos-JNK and total JNK levels were significantly reduced in siJNK-transfected cells compared with siEGFP control (Fig. 6a). IBV-induced phosphorylation of c-Jun was also lower in JNK-knockdown cells compared with siEGFP control, suggesting a reduction of JNK activity. Notably, IBV-induced PARP cleavage in siEGFP control at 20 and 22 hpi, which was partially reduced in JNK-knockdown cells of the same time point. Interestingly, compared with mock-infected cells, Bcl2decreased during IBV infection in both siEGFP and siJNK-transfected cells. However, compared with siEGFP control of the same time point, a higher Bcl2 level was always detected in the JNK-knockdown cells. JNK knockdown did not significantly affect IBV replication, as indicated by similar IBV N level compared to siEGFP control. Similarly, transfection of siJNKreduced phos-JNK and phos-c-Jun levels in IBV-infected Huh-7 cells (Fig. 6b). Knockdown efficiency was lower in Huh-7 than in H1299, as considerable amount of phos-JNK and total JNK still remained detectable. Nonetheless, JNK knockdown still partially reduced IBV-induced PARP cleavage and modulated Bcl2, similar to what happened in H1299 cells. Thus, JNK-knockdown suppressed IBV-induced apoptosis, possibly by upregulating or stabilizing Bcl2.

**Fig. 7:**
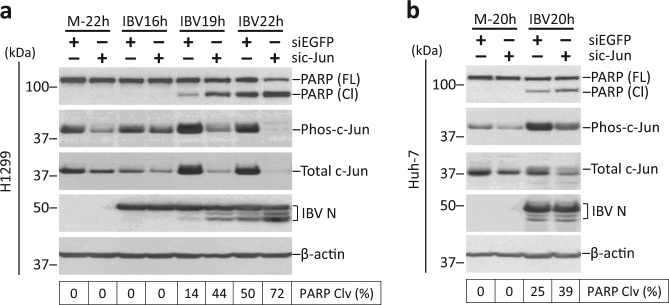
Knockdown of c-Jun potentiates IBV-induced apoptosis **a** H1299 cells were transfected with siEGFP or sic-Jun before being infected with IBV at MOI~2 or being mock infected. The cells were harvested at indicated time points and were subjected to Western blot analysis using the indicated antibodies. Beta-actin was included as loading control. Percentage of PARP cleavage was determined as in Fig. 3b. The experiment was repeated three times with similar results, and the result of one representative experiment is shown. **b** Huh-7 cells were transfected and infected as in **a.** Western blot analysis and quantification of PARP cleavage was performed as in **a.** The experiment was repeated three times with similar results, and the result of one representative experiment is shown.

H1299 cells were also transfected with siEGFP or sic-Jun before infected with IBV. Both phos-c-Jun and total c-Jun was reduced in c-Jun-knockdown cells, as compared with siEGFP control (Fig. 7a, b). Surprisingly, although knockdown of c-Jun did not significantly affect IBV replication, a significantly higher percentage of PARP cleavage was detected in c-Jun-knockdown cells compared with siEGFP control of the same time point. So, unlike JNK, c-Jun appeared to protect infected cells from apoptosis during IBV infection.

### Overexpression of Bcl2 inhibits IBV-induced apoptosis

**Fig. 8:**
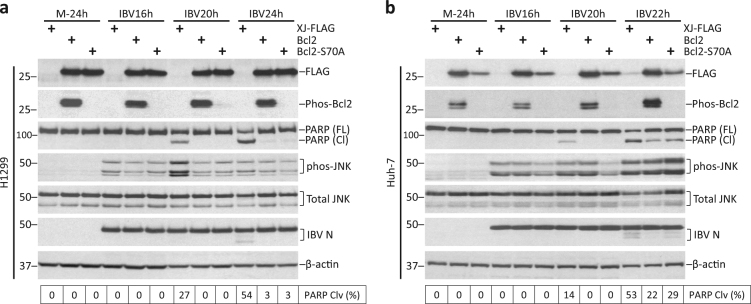
Overexpression of Bcl2 inhibits IBV-induced apoptosis **a** H1299 cells were transfected with pXJ40-FLAG, pXJ40-FLAG-Bcl2, or pXJ40-FLAG-Bcl2(S70A) before being infected with IBV at MOI~2 or being mock infected. The cells were harvested at indicated time points and were subjected to Western blot analysis using the indicated antibodies. Beta-tubulin was included as loading control. Sizes of protein ladders in kDa were indicated on the left. PARP cleavage was determined as in Fig. 3b. The experiment was repeated three times with similar results, and result of one representative experiment is shown. **b** Huh-7 cells were transfected and infected as in **a.** Western blot analysis and quantification of PARP cleavage was determined as in **a.** The experiment was repeated three times with similar results, and the result of one representative experiment is shown.

JNK was shown to directly phosphorylate Bcl2 at Ser70 to inhibit its anti-apoptotic activity in pacilitaxel-treated cells^15^. As high Bcl2 level associated with reduced apoptosis in JNK-knockdown cells, we hypothesized that a similar mechanism may function in IBV-infected cells. Wild-type Bcl2 was cloned and S70A mutant was generated, where Ser70 was mutated into alanine. H1299 cells were transfected and infected with IBV. Expression of FLAG-Bcl2 and FLAG-Bcl2-S70A was detected at similar level by Western blot (Fig. 8a). Phos-Bcl2(Ser70) was detected only in cells transfected with wild-type Bcl2, but not in the vector-transfected or S70A-transfected cells, indicating that endogenous Bcl2 was not prominently phosphorylated at Ser70. Notably, high phos-Bcl2(Ser70) level was detected in FLAG-Bcl2-transfected mock-infected cells without detectable JNK phosphorylation, suggesting that phosphorylation of ectopic Bcl2 was not dependent on IBV replication or JNK activation, and might rather be mediated by other kinase(s). IBV infection triggered significant PARP cleavage at 20 hpi and 24 hpi in vector control. In cells transfected with FLAG-Bcl2 or FLAG-Bcl2(S70A), PARP cleavage was completely abolished at 20 hpi and significantly reduced at 24 hpi. There was no significant difference in PARP cleavage between Bcl2- and Bcl2(S70A)-transfected cells of the same time point.

In Huh-7 cells, Bcl2(S70A) expression was lower than wild-type Bcl2, possibly because phosphorylation at Ser70 stabilized Bcl2 in this cell line (Fig. 8b). Similar to H1299, phosphorylation of endogenous Bcl2 was not observed, while phosphorylation of ectopic Bcl2 was detected in both mock-infected and IBV-infected cells. Overexpression of Bcl2 or Bcl2(S70A) inhibited PARP cleavage induced by IBV infection, as compared with vector control. Compared with Bcl2-transfected cells, a slightly higher PARP cleavage was observed in Bcl2(S70A)-transfected cells at 24 hpi, possibly due to its lower ectopic Bcl2 level. In summary, IBV infection did not induce detectable phosphorylation of endogenous Bcl2. Although ectopic Bcl2 was phosphorylated at Ser70, it was not mediated by JNK and did not contribute to anti-apoptotic activity during IBV infection.

## Discussion

Previous studies on coronavirus-induced JNK activation focused on overexpression of individual viral proteins^21–23, 26^. Expression levels of these proteins could be quite different in an actual infection. Moreover, interactions between viral proteins could not be recreated using the over-simplified overexpression approach. In two studies, JNK phosphorylation was detected in cells infected with MHV^19^ and SARS-CoV^20^. However, activation of upstream kinases and downstream substrates was not determined, leaving the complete pathway largely unexplored.

In this study, we used IBV as a model and characterized MKK/JNK/c-Jun pathway activation in a time course infection. We found that IBV-induced phosphorylation of MKK7, JNK, and c-Jun in the infected cells. Transfection of MKK7, but not MKK4, promoted IBV-induced phosphorylation of JNK and c-Jun. Using various MKK7 mutants, we showed that both ATP-binding activity and phosphorylation sites in MKK7 were required for optimal JNK activation in IBV-infected cells. Finally, gain-of-function and loss-of-function studies demonstrated that JNK phosphorylation plays a pro-apoptotic role during IBV infection. Thus, this study established a crucial involvement of JNK in regulating apoptosis induced by coronavirus infection.

Previous studies used SP600125 at 10–40 µM to inhibit JNK phosphorylation in coronavirus-infected cells^20, 22, 26, 31^. However, our data showed that SP600125 above 10 µM inhibited IBV replication in a dosage-dependent manner. In fact, SP600125 inhibits replication of poxviruses^32^ and VSV^33^, and non-specifically inhibits entry of HCV^34^. With the antiviral activity of SP600125 for coronavirus remaining uncharacterized, cautions should be taken to interpret these inhibitor studies.

Studies on the involvement of JNK in coronavirus-induced apoptosis are limited. In one study, JNK inhibition was found to suppress apoptosis induced by overexpression of SARS-CoV protein 6^35^. Previously we reported that hyper-phosphorylation of JNK was associated with increased apoptosis observed in IRE1-knockdown cells infected with IBV^36, 37^. Here we showed that overexpression of MKK7 or constitutively active JNK-promoted IBV-induced apoptosis, while inhibition or knockdown of JNK suppressed apoptosis, further supporting the pro-apoptotic role of JNK during IBV infection. Interestingly, our data suggested that, opposite to JNK, c-Jun actually promotes survival during IBV infection. This is not totally unexpected, because under certain situations the pro-apoptotic activities of JNK was mediated independent of c-Jun^38^. For example, both vinblastine and taxol induce JNK-dependent apoptosis with similar kinetics and induce c-Jun expression, but c-Jun phosphorylation is only observed in cells treated with vinblastine, but not taxol^39^. Also, DNA damage-induced apoptosis is mainly mediated by JNK-dependent phosphorylation of p53 or p73^9, 10^. Thus, JNK-mediated apoptosis during IBV infection might also involve similar c-Jun-independent mechanisms.

JNK can translocate into mitochondria and modify Bcl2 family proteins^7^. We showed that Bcl2 was upregulated in JNK-knockdown cells infected with IBV. Overexpression of Bcl2 or other anti-apoptotic Bcl2 family proteins can protects cells from coronavirus-induced apoptosis^40, 41^. However, our data showed that phosphorylation at Ser70 of Bcl2 was not essential for its anti-apoptotic function during IBV infection. Nonetheless, JNK might still mediate its pro-apoptotic function by indirectly modulating Bcl2 and/or other Bcl2 family proteins. Previous studies showed that JNK signaling is necessary for maintenance of persistent SARS-CoV infection in cells^25, 42^. Establishment of persistent infection was observed after the apoptotic events, so it is possible that JNK first serves as a pro-apoptotic protein in the acute phase of SARS-CoV infection, but switches to a pro-survival factor in persistently infected cells.

The IBV strain we used has been passaged in Vero cells for 65 times^43^, but it can still effectively infect chicken fibroblast cell line DF1^44^. DF1 is not used here because phos-JNK antibody could not detect the expression of avian homolog(s) (data not shown). Also, the cleaved form of PARP could not be strongly detected by PARP antibody. Several studies by our group on stress response have used H1299 and Huh-7 cells^18, 28^. To be consistent with these previous experiments and to make study on signaling cross-talk between pathways possible, we chose to use the same cell lines in this study.

Cellular response against different virus infections can be substantially different. Within the coronavirus family, JNK is required for persistent SARS-CoV infection in Vero E6 cells^20^, has no effect on apoptosis induced by porcine epidemic diarrhea virus^45^, and promotes IBV-induced apoptosis in this study. Since the outcomes are so dramatically different, we consider it scientifically important to investigate in detail the activation of JNK pathway induced by IBV, a prototype gammacoronavirus. Unlike previous studies that rely on transfection of viral proteins, we examined the activation of JNK pathway in the context of an actual infection. Moreover, to our knowledge, this is also the first study to investigate potential involvement of JNK in coronavirus-induced apoptosis.

Virus-induced apoptosis is an important event that determines virulence and pathogenicity. At early infection, apoptosis can serve as an antiviral strategy, but apoptosis induced at late stage of infection may facilitate virus spreading and immune evasion. Therapeutical manipulation of JNK may be a viable approach to control viral replication or tissue damage associated with infection, as shown in a recent study for dengue virus^46^. Also, vaccine strains that induced reduced apoptosis may be desirable, because viral epitopes are displayed longer for immune stimulation, favoring activation of cell-mediated immune response. IBV infection leads to a significant economic loss to poultry industry worldwide, with new variants emerging every year. A deeper understanding of how this important virus interacts with host cells, including the JNK pathway, provides new guidelines for construction of more suitable viral and cell systems for vaccine development.

## Materials and methods

### Virus and cell lines

The egg-adapted Beaudette strain of IBV (ATCC VR-22) was obtained from American Type Culture Collection (ATCC) and adapted to Vero cells as described^43^. To prepare the virus stock, monolayers of Vero cells were infected at a multiplicity of infection (MOI) of ~0.1 and cultured in plain Dulbecco Modified Eagle Medium (DMEM, Gibco) at 37 °C for 24 h. After three freeze/thaw cycles, cell lysate was clarified by centrifugation at 1500 × *g* at 4 °C for 30 min. The supernatant was aliquot and stored at −80 °C as virus stock. The titer of the virus stock was determined by plaque assays. Mock lysate was similarly prepared by subjecting uninfected Vero cells to three freeze/thaw cycles and clarified by centrifugation.

Inactivation of IBV was performed by exposing the virus stock to 120,000 mJ/cm^2^ of 254-nm shortwave UV radiation for 15 min with a CL-1000 cross-linker (UVP)^47^. To demonstrate that IBV had been inactivated, the Vero cells were incubated with UV-IBV and the cell lysates were analyzed by Western blot to confirm that no viral proteins can be detected.

H1299 cells were cultured in RPMI 1640 medium (Gibco) supplemented with 5% fetal bovine serum (FBS) and 1% penicillin-streptomycin (Gibco). All cells were grown in a 37 °C incubator supplied with 5% CO_2_. Huh-7 cells were cultured in DMEM supplemented with 10% FBS and 1% penicillin-streptomycin (Gibco). In all the experiments, cells were washed twice with serum-free medium before infected with IBV at an MOI of ~2 or incubated with equal volume of UV-IBV in serum-free medium. After 2 h of absorption, the cells were washed twice with serum-free medium and incubated at 37 °C before harvested.

### Antibodies, chemicals, and reagents

The antibodies against β-actin (#4967), FLAG-tag (#2044), PARP (#9532), total JNK (#9252), phospho-JNK (#9251), total c-Jun (#9165), phospho-c-Jun (#9261), Total MKK4 (#9152), phospho-MKK4 (#9151), Total MKK7 (#4172), phospho-MKK7 (#4171), total Akt (#4691), phospho-Akt (#4060), total Bcl2 (#2876), and phospho-Bcl2 (#2827), were purchased from Cell Signaling Technology. The anti-serum against IBV S protein and N protein were from rabbits immunized with bacterial expressed fusion proteins as previously described^48, 49^.

### Plasmid constructions and transfection

The complementary DNA(cDNA) of human MKK4 (RefSeq NM_003010) was amplified from total RNA of H1299 cells by reverse transcription-polymerase chain reaction (RT-PCR) using the forward primer 5′-CCCGGATCCATGGCGGCTCCGAGCCCGAG-3′ and reverse primer 5′-CTTGGTACCTCAATCGACATACATGGGAGAGCTGGGAG-3′. The cDNA of human MKK7 (RefSeq NM_145185) was amplified similarly using the forward primer 5′-CCCGGATCCATGGCGGCGTCCTCCCTGGAAC-3′ and reverse primer 5′-CTTGGTACCCTACCTGAAGAAGGGCAGGTGGGG-3′. The PCR products were digested and inserted into *pXJ40-FLAG* at the *BamHI* and *KpnI* restriction sites. The resulting constructs were named *pXJ40-FLAG-MKK4* and *pXJ40-FLAG-MKK7*, respectively. The cDNA of human Bcl2 (RefSeq NM_000633) was amplified from total RNA of H1299 cells by RT-PCR using the forward primer 5′-CGCAGATCTGCGCACGCTGGGAGAACAGGGTAC-3′ and reverse primer 5′-GCCGCTCGAGTCACTTGTGGCCCAGATAGGCACC-3′. The PCR product was digested and inserted into *pXJ40-FLAG* at the *BamHI* and *XhoI* sites to generate *pXJ40-FLAG-Bcl2*.

The ATP-binding mutant of MKK7-KM was generated by site-directed mutagenesis to replace amino acid lysine 149 with methionine in *pXJF40-FLAG-MKK7* using the forward primer 5′-CATTGCCGTTATGCAAATGCGGC-3′ and reverse primer 5′-GCCGCATTTGCATAACGGCAATG-3′. The MKK7-3E mutant was generated by replacing amino acids serine 271, threonine 275, and serine 277 with glutamic acid using forward primer 5′-GAGAAAGCCAAGGAGCGGGAAGCCGGCTGTGCCGCC-3′ and reverse primer 5′-TTCCCGCTCCTTGGCTTTCTCGTCCACCAGGCGGCC-3′. The MKK7-3A mutant was generated by replacing the same sites with alanine using forward primer 5′-GCCAAAGCCAAGGCGCGGGCCGCCGGCTGTGCCGCC-3′ and reverse primer 5′-GGCCCGCGCCTTGGCTTTGGCGTCCACCAGGCGGCC. The serine 70 in *pXJ40-FLAG-Bcl2* was replaced with alanine using forward primer 5′-GTCGCCAGGACCGCGCCGCTGCAGAC-3′ and reverse primer 5′-GTCTGCAGCGGCGCGGTCCTGGCGAC-3′ to generate the Bcl2-S70A mutant. The constructs *pcDNA-FLAG-MKK7-JNK1* and *pcDNA-FLAG-MKK7-JNK1(APF)* were obtained from Addgene as previously descried^29^.

Transfection of plasmids DNA was performed using Lipofectamine 2000 reagent (Invitrogen) according to the manufacturer’s instructions. Briefly, the cells were plated to a 12-well plate the day before transfection. For each well, 0.8 µg of plasmid DNA and 2 µl of Lipofectamine 2000 were each diluted with 100 µl of plain medium and incubated for 5 min. Then the diluted plasmid and transfection reagent were mixed by brief vortex and incubated for another 20 min. The H1299 cells were changed with 800 µl of medium containing 5% FBS and the transfection mixture was added to each well. The cells were incubated at 37 °C for 6–8 h before replacing with complete medium. At 24 h post-transfection, the cells were infected with IBV at an MOI of 2 or were mock infected, and were continued to be incubated before being harvested for protein and/or RNA analysis at indicated time points.

### RNA interference

JNK1/2 siRNA (+): 5′-AAAGAAUGUCCUACCUUCU dTdT-3′^50^ and control EGFP siRNA (+): 5′-GCUGACCCUGAAGUUCAUC dTdT-3′ were purchased from Sigma. Transfection of siRNA was performed using DhamaFECT2 transfection reagent (Dharmacon, Thermo Fisher Scientific) according to the manufacturer’s instructions. At 48 h post-transfection, the cells were infected with IBV at an MOI of 2 or were mock infected, and were continued to be incubated before being harvested for protein analysis at the indicated time points.

### SDS-PAGE and Western blot analysis

Cells were infected with IBV and harvested at indicated times points using cell scrapers (Corning). After centrifugation at 16,000 × *g* for 1 min, the supernatant was discarded and the pellets were lysed in 1× RIPA buffer. After clarifying by centrifugation and determination of protein concentration by spectrophotometer, the cell lysates were mixed with Laemmli sample buffer containing 100 mM dithiothreitol^51^. The protein samples were boiled at 90 °C for 5 min and centrifuged at 16,000 × *g* for 5 min. Equal amount of protein samples were subjected to sodium dodecyl sulfate-polyacrylamide gel electrophoresis (SDS-PAGE) and transferred to 0.2 µm nitrocellulose membranes (Bio-Rad). After the nonspecific antibody binding sites were blocked with 5% skim milk in Tris-buffered saline (20 mMTris-HCl pH 7.4, 150 mMNaCl) containing 0.1% Tween 20, the membranes were incubated with 1 µg/ml primary antibodies at 4 °C overnight. After washing with Tris-buffered saline, the membranes were incubated with 1:2000 diluted anti-mouse or anti-rabbit IgG antibodies conjugated with horseradish peroxidase (DAKO) at room temperature for 2 h. The membranes were washed and the proteins detected with a chemiluminescence detection kit (Amersham Biosciences) and medical X-ray films (Fujifilm) according to the manufacturer’s instructions. The films were scanned as gray scale 8-bit images and the density of bands were determined by the NIH software ImageJ. All experiments were repeated for at least three times with similar result, and one of the representative results is shown.

### Virus titration

Cell-free supernatants of IBV-infected cells collected at different time points were clarified by centrifugation and 10-fold serially diluted using serum-free DMEM. The viral titers were determined by plaque assay. Briefly, 250 µl of diluted supernatants were applied to confluent monolayers of Vero cells in 6-well plates. The plate was agitated every 10 min to ensure proper coverage of the monolayers. After 2 h of adsorption, unbound viruses were removed and cells were washed twice with DMEM. A total of 2 ml overlay medium (0.4% agarose in DMEM) was added to each well and the plates were incubated at 37 °C for 2 days before plaques formed. Agarose overlay was removed and the cells were fixed with 4% formaldehyde before staining with crystal violet. Finally plaque numbers were counted and the titers of individual samples were expressed in the unit of logarithm of plaque-forming units per ml. Each sample was titrated in triplicate in each experiment.
